# Expression Characterization of ABCDE Class MADS-Box Genes in *Brassica rapa* with Different Pistil Types

**DOI:** 10.3390/plants12112218

**Published:** 2023-06-04

**Authors:** Yi Zhang, Tong Zhao, Yuqi Wang, Rong Yang, Weiqiang Li, Kaiwen Liu, Nairan Sun, Iqbal Hussian, Xinyan Ma, Hongrui Yu, Kun Zhao, Jisuan Chen, Xiaolin Yu

**Affiliations:** 1 Department of Horticulture, College of Agriculture and Biotechnology, Zhejiang University, Hangzhou 310058, China; 21916136@zju.edu.cn (Y.Z.); 12216071@zju.edu.cn (T.Z.); 22116200@zju.edu.cn (Y.W.); 3140100056@zju.edu.cn (R.Y.); 12216070@zju.edu.cn (W.L.); 12016052@zju.edu.cn (K.L.); 22016168@zju.edu.cn (N.S.); 11816119@zju.edu.cn (I.H.); 22016160@zju.edu.cn (X.M.); 22116202@zju.edu.cn (H.Y.); 11816013@zju.edu.cn (K.Z.); 2 Group of Vegetable Breeding, Hainan Institute of Zhejiang University, Sanya 572000, China; 3 Ningbo Haitong Food Technology Co., Ltd., Ningbo 315300, China; cjs@haitonggroup.com; 4 Key Laboratory of Horticultural Plant Integrative Biology Research and Application in Zhejiang Province, Hangzhou 310058, China

**Keywords:** MASD-box genes, ABCDE model, tissue specificity, flower development, multilocular silique, Chinese cabbage, *Brassica rapa*

## Abstract

MADS-box is a vital transcription factor family that functions in plant growth and development. Apart from *APETALA2*, all genes in the ABCDE model that explain the molecular mechanism of floral organ development belong to the MADS-box family. Carpel and ovule numbers in plants are essential agronomic traits that determine seed yield, and multilocular siliques have great potential for the development of high-yield varieties of *Brassica*. In this study, ABCDE genes in the MADS-box family from *Brassica rapa* were identified and characterized. Their tissue-specific expression patterns in floral organs and their differential expression in different pistil types of *B*. *rapa* were revealed by qRT-PCR. A total of 26 ABCDE genes were found to belong to the MADS-box family. Our proposed ABCDE model of *B*. *rapa* is consistent with that of *Arabidopsis thaliana*, indicating that ABCDE genes are functionally conserved. These results of qRT-PCR showed that the expression levels of class C and D genes were significantly different between the wild-type (wt) and tetracarpel (*tetrac*) mutant of *B*. *rapa*. Interestingly, the expression of the homologs of class E genes was imbalanced. Therefore, it is speculated that class C, D, and E genes are involved in developing the carpel and ovule of *B*. *rapa*. Our findings reveal the potential for the selection of candidate genes to improve yield traits in *Brassica* crops.

## 1. Introduction

Plant development is an orderly and complex process, with the flower as the most complex organ structure. Floral organ development is a crucial stage in the plant life cycle, which is not only affected by the external environment, such as temperature, light, water, and gas conditions, but also regulated by internal factors, such as hormones and genes [1,2]. Floral morphogenesis is controlled by fewer genes, which provides a molecular basis for the study of evolutionary change among plant reproductive structures [1]. Coen and Meyerowitz [3] formulated the famous ABC model of flower development by analyzing mutations affecting flower structure in *Antirrhinum majus* L. and *Arabidopsis thaliana*. On this basis, the ABCDE (or AE model) model was finally established by extensive research on mutants with floral organ identity defects [4,5,6,7]. Briefly, according to this model, the development of sepals, petals, stamens, carpels, and ovules is controlled by A+E genes, A+B+E genes, B+C+E genes, C+E genes, and D+C+E genes, respectively, in which class A genes and class C genes antagonize each other [5,8]. All genes involved in the ABCDE model belong to the MADS-box gene family except for *APETALA2* (*AP2*) [9,10].

MADS-box family genes contain a conserved MADS-box motif that encodes the domain of the transcription factors responsible for nuclear localization, DNA binding, dimerization, and accessory factor binding [11]. Based on phylogenetic relationships, the plant MADS-box gene family can be divided into two major types, referred to as type I and type II. Type I MADS-box genes can be further divided into Mα, Mβ, and Mγ classes, while type II genes can be subdivided into MIKC^C^ and MIKC* classes [12]. 

MADS-box genes play important roles in plant biological processes, especially in flower development.

Class A genes

It has been described that mutations of *APETALA1* (*AP1*) in the class A genes of *A. thaliana* cause the partial conversion of flowers into inflorescence shoots [13], and this gene is involved in sepal and petal identity specification as well as bract suppression in flowers [14].

Class B genes

Class B genes in *A. thaliana* include *APETALA3* (*AP3*) and *PISTILLATA* (*PI*) [15,16,17,18,19]. Ma, et al. [20] collected data from chimeric constructs of transgenic *A. thaliana* plants, which suggested that the MADS domain was irreplaceable for the function of *IiAP3* in *Isatis indigotica*, and the K domain of *IiAP3* was involved in the specific identification of stamens. In addition, the K domain of *IiPI* was mainly related to the formation of petals, and the C-terminal region of *IiPI* was involved in characterizing stamens.

Class C genes

*AGAMOUS* (*AG*) is a class C gene associated with flower development in *A*. *thaliana* [3,15]. *TeAG1*, a class C gene of *Tagetes erecta*, was shown to play a role in stamen and carpel identity, and it led to curling rosette leaf and early flowering in *A*. *thaliana* [21]. Gómez-Felipe, et al. [22] generated a model of a gene regulatory network in *A*. *thaliana*, in which cytokinin signaling was mainly upstream and parallel to AG activity. Cytokinin induced carpelloid features in an AG-dependent manner and the expression of the transcription factors *CRC*, *SHP2* and *SPT* that are involved in carpel development.

Class D genes

*STK*, *SHATTERPROOF1* (*SHP1*), and *SHATTERPROOF2* (*SHP2*) in *A*. *thaliana* belong to class D genes [7,9]. *OsMADS13*, the rice (*Oryza sativa* L. ssp. *japonica*) ortholog of STK, functions in floral meristem determinacy. Mutants of *OsMADS13* were female sterile and ovules were converted into carpelloid structures [23]. Di Marzo, et al. [24] described the regulation of pollen tube development by *CESTA* (*CES*) and *STK*. *STK* is co-expressed with *CES*, a basic Helix-Loop-Helix (bHLH) transcription factor-encoding gene. The *stk ces*-*4* mutants had reduced ovule fertilization, which was due to a defect in carpel fusion that resulted in the formation of holes at the center of the septum where the transmitting tract differentiates.

Class E genes

Class E genes include *SEPALLATA1* (*SEP1*), *SEP2*, *SEP3*, and *SEP4* [8,25,26]. The constitutive expression of *IiSEP2*, a homologous gene of *SEP2*, in the Columbia (Col-0) ecotype of *A*. *thaliana* led to early flowering and a reduced number of flowers and floral organs. Due to the overexpression of *IiSEP4* in Landsberg *erecta* (L*er*) of *A. thaliana*, sepals were converted into curly carpelloid structures accompanied by the generation of ovules [27,28]. In addition, Zhang, et al. [29] supposed that *VvMADS39*, a class E MADS-box gene of *Vitis vinifera* orthologous to *A*. *thaliana SEP2*, may regulate the development and contribute to the formation of seedless fruit.

*Brassica rapa*, as a member of the cruciferous family, is rich in crude fiber, vitamins, and carotene. Due to its high yield and nutritional value, it has become one of the most important leafy vegetables and rapeseed sources grown in the world. The carpel is the female reproductive organ of flowering plants (angiosperms) that encloses the ovules and develops into fruits. It is suggested to be the most important autapomorphy of angiosperms [30]. Ovules are the precursor of seeds, growing on the placenta on the inner wall of the ovary through the peduncle. Ovule primordium initiation is a prerequisite for seed formation, which determines the maximum possibility of ovule number in each flower and greatly influences seed yield [31]. In general, the number of carpels in plants is stable due to genetic regulation; however, it changes in some plants during development, which in turn modifies the number of ovules, resulting in different seed yields [32]. Therefore, it is necessary to understand the factors that control carpel numbers and ovule initiation. Unfortunately, very little is known about carpel and ovule development in *B*. *rapa*. Wang, et al. [33] completed the sequencing of the whole genome of *B. rapa* in 2011, which has facilitated the analysis of the molecular mechanisms regulating the development of carpel and ovule in this species.

Herein, we selected ABCDE genes in the MADS-box family, which were related to floral organ development in *B*. *rapa*, from a previous study by Saha, et al. [34], and established an ABCDE model of flower development. We demonstrated that class C, class D, and some class E genes are highly expressed in the pistil and ovule of *B*. *rapa*. Further investigations revealed the different expression patterns of these three classes of genes in two pistil types of *B*. *rapa*, and the expression of the homologs of class E genes was imbalanced. Our results indicated that class C, D, and E genes are involved in the development of pistil and ovule in *B*. *rapa*, and the expression dosage of class E homologous genes may affect the pistil and ovule traits. In conclusion, we described the expression characteristics of ABCDE MADS-box genes in *B*. *rapa* flower development, analyzed the differences of these genes between the wt and *tetrac*, and finally, screened out the target genes that may cause the mutation of multilocular silique. This study can provide clues for increasing the ovule number and seed yield in *Brassica* crops.

## 2. Results

### 2.1. Phenotypic Characterization of Different Pistil Types of B. rapa

**Figure 1:**
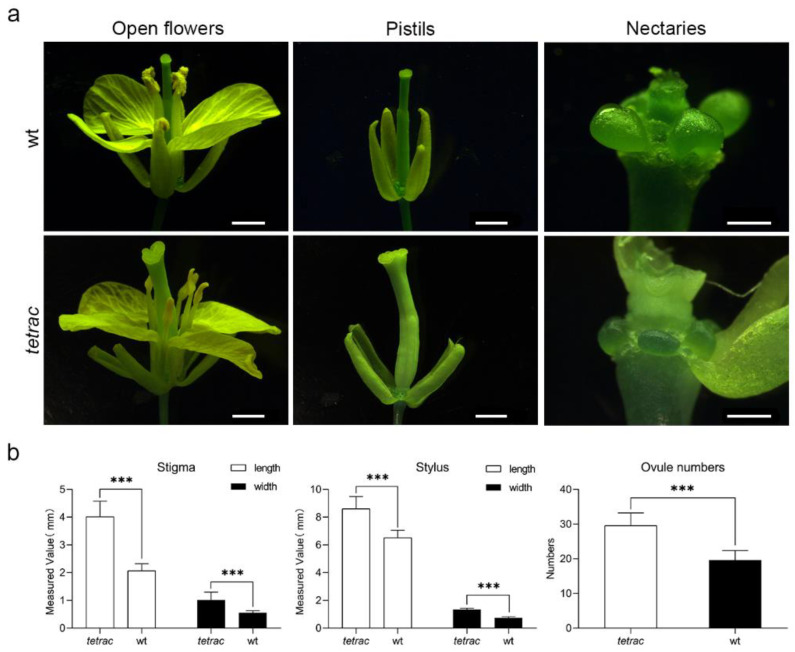
Floral organs of two pistil types of *Brassica rapa*, wt and *tetrac*. (**a**) Phenotypic observation of wt and *tetrac* flower organs by using a stereomicroscope, the bars of open flowers and pistils = 2 mm, and the bars of nectaries = 0.5 mm; (**b**) Statistical data of pistil (stigma and stylus) and ovule numbers of wt and *tetrac*; the data shown are mean ± SD of at least three biological replicates. Significant differences between wt and *tetrac* determined by an independent Student’s *t*-test are indicated with *** (*p* < 0.001).

The phenotypic observation of floral organs of different pistil types of *B*. *rapa*, wt and *tetrac*, was carried out by stereomicroscope. The flowers of wt consist of four whorls of floral organs, including four sepals, four petals, six stamens, four nectaries, and two congenitally fused carpels that form the central pistil. Obviously, most of the floral organs of *tetrac* are larger than those of wt, and its pistil is formed by four fused carpels. However, the nectary sizes of the wt flower are larger than those of *tetrac*. (Figure 1a). 

A vernier caliper was employed to measure the differences in pistils between wt and *tetrac* in more detail. The results showed that flowers of *tetrac* had a significantly larger stigma and stylus, and the number of ovules in plant of tetrac also much more than that of wt (Figure 1b and Table S1).

### 2.2. Identification and Chromosomal Localization of ABCDE Genes in MADS-box Gene Family in B. rapa

A total of 167 MADS-box genes were identified from the entire *B. rapa* genome, among which 26 ABCDE genes belonged to MIKC^C^ classes, including 3 class A genes, 5 class B genes, 2 class C genes, 6 class D genes, and 10 class E genes [34].

**Figure 2:**
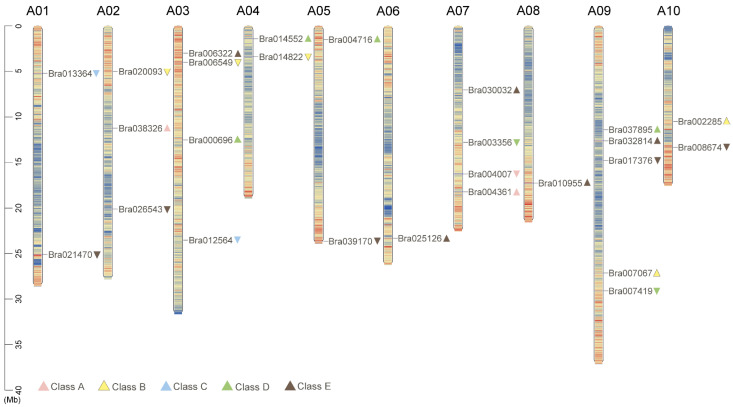
Chromosome distribution of 26 ABCDE class MADS-box genes in *B. rapa*. The different colors of the triangles indicate different types of MADS-box genes. The direction of the top corner of the triangle indicates the encoding direction of the gene: the downward direction is forward encoding, and the upward direction is reverse encoding.

The physical mapping of identified ABCDE genes in the MADS-box family in *B*. *rapa* revealed that all of the target genes were distributed across all chromosomes, among which chromosome A09 contained the most ABCDE genes (5), whereas chromosomes A06 and A08 contained the fewest (1). In addition, there were 13 forward-coding genes and 13 reverse-coding genes among the 26 ABCDE genes (Figure 2).

### 2.3. Gene Structure and Conserved Motif Analysis

To investigate the evolutionary relationship of ABCDE genes in the MADS-box family in B. rapa, we constructed a phylogenetic tree using the Maximum likelihood algorithm. The results showed that each ABCDE gene in *A*. *thaliana* corresponds to 1–3 homologous genes in *B*. *rapa*, indicating that gene amplification occurred in the process of genome evolution in *A*. *thaliana* and *B*. *rapa*.

**Figure 3:**
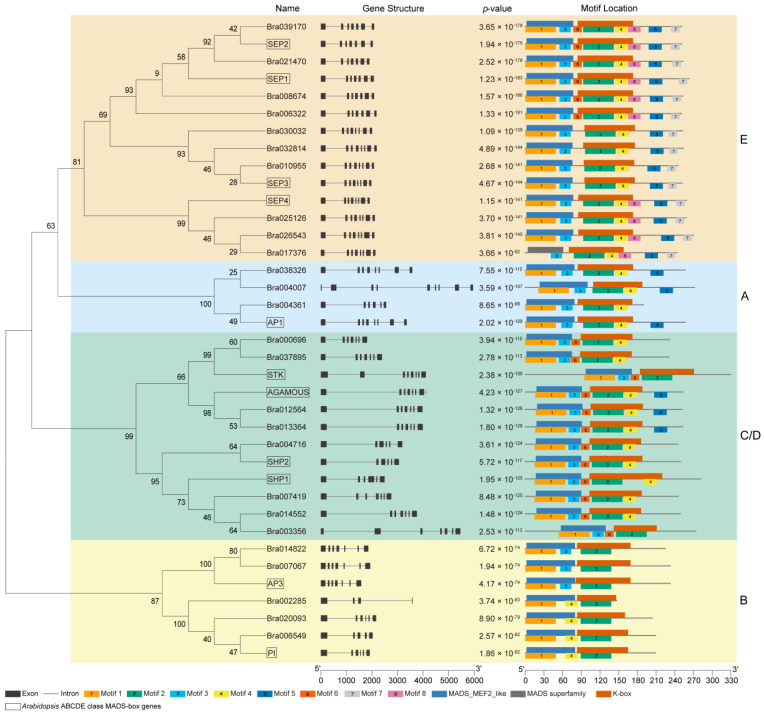
Phylogenetic relationships, gene structure, conserved motif compositions and structural domains of *B. rapa* and *Arabidopsis* ABCDE class MADS proteins. The maximum likelihood tree was constructed with the aligned protein sequences of *B. rapa* and *Arabidopsis* ABCDE class MADS-box genes. Eight motifs were identified and displayed in different colors. The combined match *p*-value is defined as the probability that a random sequence (with the same length and conforming to the background) would have position *p*-values such that the product is smaller or equal to the value calculated for the sequence under test.

To assess the diversity and similarity of *B*. *rapa* ABCDE genes, the intron/exon arrangements and conserved motifs were analyzed according to their phylogenetic relationship (Figure 3). Class A genes contained 5–8 introns, class B genes contained 3–6 introns, class C genes contained 6 introns, class D genes contained 5–6 introns, and class E genes contained 6–7 introns. A lower similarity was found in the structure of class A genes, which showed a difference in the length of the intron, whereas the structure of class E genes was the most similar.

To explore the conserved motifs in the ABCDE genes in *B*. *rapa*, the MEME motif search tool was employed. A total of eight motifs were identified and were named as Motif 1 to Motif 8, respectively. ABCDE genes in the same class had similar motifs (Figure 3 and Table S2). Class B genes showed the simplest protein structure with only 3 motifs, whereas class E genes presented a more complex protein structure with 6–8 motifs. Among all these eight motifs, motifs 1 and 3 represent the MADS-domain and the main domain motif 1 was found in most genes except for *Bra017376*. Motifs 2 and 4, specifying the K-box, were found in all target genes. Motif 4 was present in most genes but not B-*AP3* lineage genes and *Bra003356*. Only class A (except for *Bra004361*), C and E genes included motif 5. Motif 6 was detected in all C, D, E-*SEP1*, and E-*SEP2* lineage genes. Motif 7 was only found in class E genes, and Motif 8 was prevalent in all class E genes except for E-*SEP3* lineage genes.

### 2.4. Cis-Elements and Potential Transcription Factor Binding Sites

**Figure 4:**
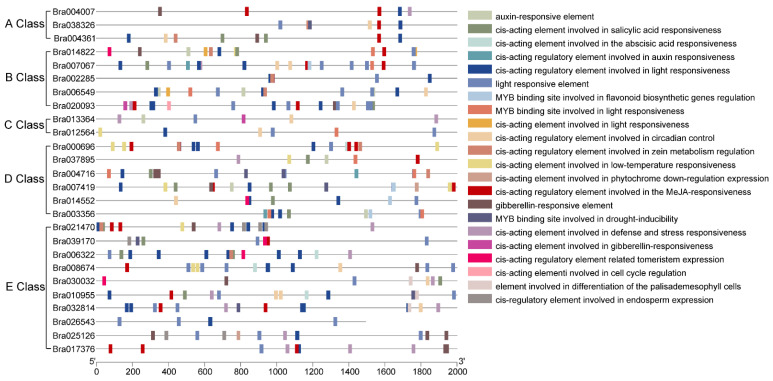
Prediction of *cis*-acting elements of the ABCDE class MADS-box gene family in *B. rapa*. The upstream 2 kb sequences of ABCDE class MADS-box genes in *B. rapa* were analyzed through PlantCARE.

Transcription factors (TFs) control and regulate gene expression by binding with cis-elements in the promoters of target genes. To investigate the regulatory gene networks of ABCDE genes in the MADS-box family, the cis-elements of the upstream 2000-bp sequences of BrMADS were predicted using the PlantCARE database. A total of 28 biological processes were annotated to 26 target genes (Figure 4 and Table S3). Of note, light-responsive boxes existed in all ABCDE genes. Hormone-responsive cis-elements were found in most genes. The cell cycle regulation cis-element (MSA-like) was only found in *Bra020093*. The endosperm expression element (GCN4_motif) and palisade mesophyll cells differentiation element (HD-Zip 1) only existed in class E genes (Table S4). 

To further identify the transcription factors that potentially control the ABCDE genes, the promoters of 26 target genes were processed using MEME software (version 5.4.1) to locate cis-motifs. Twenty enriched motifs were found, and a total of sixteen TFs along with their DNA bind sites were identified in four motifs by comparison with the JASPAR database. Nine TFs were other C4 zinc finger-type factors, and other TFs belonged to C2H2 zinc finger factors, BBR/BPC and AP2/EREBP (Table S5).

### 2.5. Proposed ABCDE Model in B. rapa

**Figure 5:**
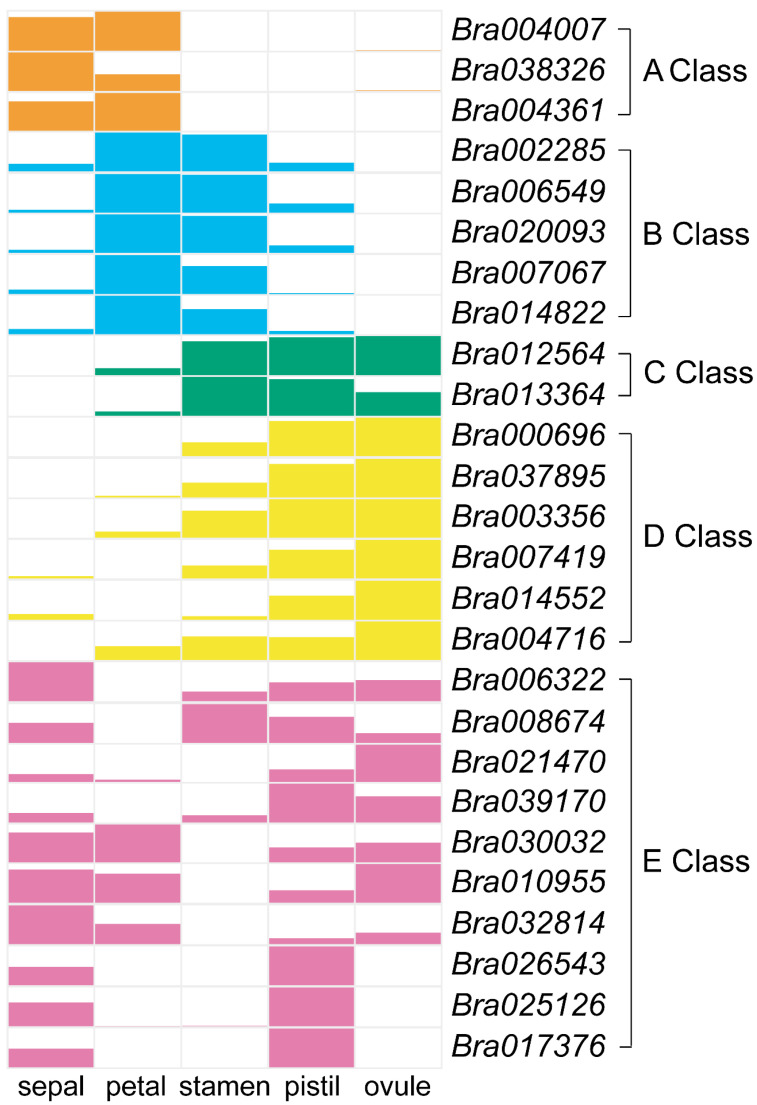
Proposed *B. rapa* flower development model based on the expression patterns and referring to the ancestral functions of homeotic MADS-box genes. Blocks in different colors with distinct heights represent different expression levels.

We analyzed the qRT-PCR data for MADS-box genes in five floral organs (sepals, petals, stamens, pistils, and ovules) to determine whether certain genes were associated with specific *B*. *rapa* floral organs (Figure 5). The ABCDE genes had typical temporal and spatial expression profiles in the five analyzed floral organs. The expression of class A genes was high in sepals and petals. B-*PI* lineage genes were highly expressed in petals and stamens, whereas *AP3* lineage genes showed lower expression in stamens. Class C genes were highly expressed in stamens, pistils, and ovules. The expression of class D genes was high in pistils and ovules but was detected at lower levels in stamens. Class E genes were detected in all floral organs, and the orthologs of E-*SEP1*, -*SEP2*, and -*SEP3* genes showed biased expression in different floral organs. The orthologs of *SEP1* (*Bra006322* and *Bra008674*) expressed in most floral organs except for the petal, while the expression of *SEP2* orthologs were high in pistil (*Bra039170*) and ovule (*Bra021470*). The *SEP3* orthologs (*Bra030032*, *Bra010955* and *Bra032814*) were more highly expressed in sepal, petal, and ovule, less expressed in the pistil, and hardly detected in the stamen. The expression of *SEP4* ortholog (*Bra026543*, *Bra025126,* and *Bra017376*) was extremely high in the pistil, but hardly detected in petal, stamen, and ovule. The ABCDE model of *B*. *rapa* was consistent with that of *A*. *thaliana*, and all classes of ABCDE genes showed redundant gene functions.

### 2.6. Differential Expression of ABCDE Genes in the MADS-Box Family in Different Pistil Types of B. rapa

To determine whether specific genes were associated with the different *B*. *rapa* pistil types, we studied the tissue-specific expression pattern of ABCDE genes in the MADS-box family during wt and *tetrac* flower development.

**Figure 6:**
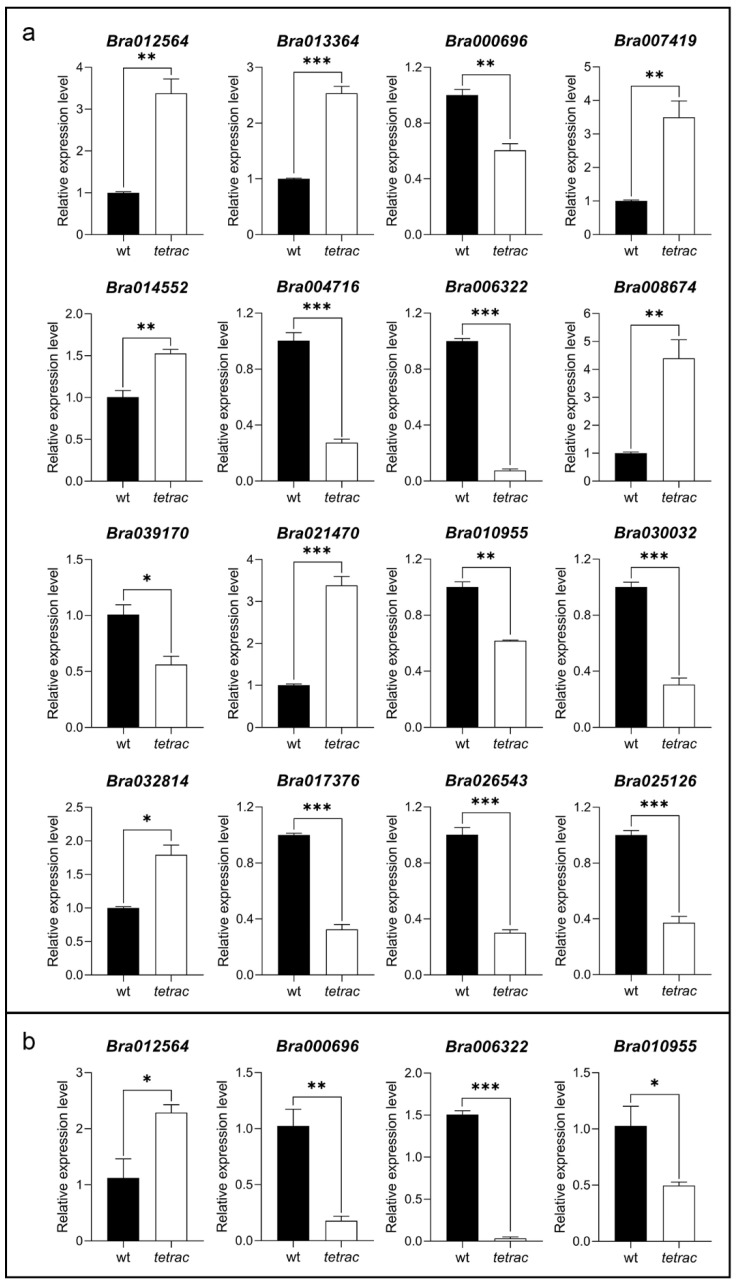
Tissue-specific expression analysis of ABCDE class MADS-box genes in the pistils (**a**) and ovules (**b**) of wt and *tetrac* based on qRT-PCR. Data shown are mean ± SD of at least three biological replicates. Significant differences between wt and *tetrac* determined by an independent sample *t*-test are indicated with * (*p* < 0.05), ** (*p* < 0.01), and *** (*p* < 0.001).

According to the above established ABCDE model, the class C, D, and E genes related to the development of pistil and ovule were focused on. Two class C genes (*Bra012564* and *Bra013364*), two D-*SHP1* lineage genes (*Bra007419* and *Bra014552*), and three genes in class E, including one *SEP1* ortholog (*Bra008674*), one *SEP2* ortholog (*Bra021470*) and one *SEP3* ortholog (*Bra032814*), showed higher expression in the pistil of *tetrac*, which might be positively associated with *B*. *rapa* pistil development. On the contrary, the expression of the following genes was lower in the pistil of *tetrac* (Figure 6a): two class D genes including a D-*STK* lineage gene (*Bra000696*) and a D-*SHP2* lineage gene (*Bra004716*), and seven class E genes, including one *SEP1* ortholog (*Bra006322*), one *SEP2* ortholog (*Bra039170*), two *SEP3* orthologs (*Bra010955* and *Bra030032*) and all *SEP4* orthologs (*Bra017376*, *Bra026543*, and *Bra025126*).

Only four genes showed differential expression between the ovules of wt and *tetrac*. *Bra012564* was highly expressed in the ovules of *tetrac*, while *Bra000696*, *Bra006322*, and *Bra010955* showed extremely high expression in the ovules of wt, indicating that they play a crucial role in the ovule development process (Figure 6b). 

## 3. Discussion

Genes in the MADS-box family are involved in floral organ development. In this study, 26 ABCDE genes in this family were identified in *B*. *rapa*, which is more than the number of ABCDE genes in *A*. *thaliana*. This difference is the result of genome replication. Whole-genome duplication, a common phenomenon in the process of plant evolution, has been observed in all plant genomes sequenced to date. It can drive the expansion of plant gene families and yield the production of new genes. *A*. *thaliana* has undergone three paleo-polyploidy events shared with *Cruciferae*, including a paleohexaploidy event (γ) and two paleotetraploidy events (β, then α) [35]. In addition, a whole-genome triplication (WGT) event is thought to have occurred in *B*. *rapa* after the *Arabidopsis*–*Brassiceae* split, dividing its genome into three subgenomes named as LF, MF1 and MF2 [36]. ABCDE genes have 1–3 orthologs in *B*. *rapa* due to WGT, which may result in their more complex functions in flower and organ development in this plant.

There are differences in gene structure among ABCDE genes in the MADS-box family, but those in the same class have a similar gene structure. In addition to the highly conserved MADS-box motif shared by all members of this gene family, ABCDE genes also contain a motif specific to them, and homologous genes contain the same motif. These data indicate that the functions of all kinds of ABCDE genes are assumed, and there may be functional redundancy among homologous genes.

In the ABCDE model in *B*. *rapa* established in this study, the ortholog of the class A gene, *AP1*, was highly expressed in sepals and petals, which was consistent with its expected function in sepal and petal identity specification. Genes in the B-*AP3* and -*PI* lineage are highly expressed in petals and stamens. 

According to the classical flower development model, C and D genes evolved from a common ancestor gene, which is attributed to the ancient gene duplication events in the evolution of the *AG* subfamily. The first duplicate event produced the C and D lineage, and the subsequent event in the C lineage resulted in the production of the *euAG* and *PLE* lineage (*SHP1*/*2*). In *A*. *thaliana*, class C and D genes have partly overlapping expression patterns but also evolved their specific functions [7,37,38]. *AG* (*euAG* lineage) is the only gene with full C function activity, which plays a role in stamen identity and carpel identity. Ovules are controlled by class D ovule identity genes *SHP1*, *SHP2*, and *STK* [7,39,40,41]. In this study, the ortholog of the class C gene, *AGAMOUS*, can be detected in the stamens, pistils, and ovules of *B*. *rapa*, indicating that they have conservative expression patterns in line with the ancestral gene functions. The D-*STK*, -*SHP1*, and -*SHP2* lineage genes were found to be preferentially expressed in the ovule of *B*. *rapa* and are also highly expressed in the pistil. In addition, we also detected a lower level of expression of these genes in the stamens of *B*. *rapa*. According to the qPCR result of wt and *tetrac*, the C-*AGAMOUS* and D-*SHP1* orthologs were highly expressed in the pistil of *tetrac*, indicating that they played a redundant positive role in increasing the number of carpels. Moreover, a higher expression level of a C-*AGAMOUS* lineage gene (*Bra012564*) was detected in the *tetrac* ovules, showing its effective role in promoting ovule development. Interestingly, the expression of other D lineage genes, *STK* and *SHP2*, decreased significantly in the pistil of *tetrac*, suggesting that they negatively regulate the development of carpels. The different expression patterns of *SHP1*, *SHP2* and *STK* in the pistil of wt and *tetrac* suggest that D lineage genes may show various degrees of subfunctionalization and neofunctionalization in regulating carpel development. In addition, a D-*STK* ortholog (*Bra000696*) negatively regulates ovule development because its expression in *tetrac* ovule is significantly down-regulated.

Phylogenetic tree analysis showed that the *SEP*-like subfamily experienced several gene duplication events in the process of evolution, and finally produced four *SEP*-like genes in *A*. *thaliana*: *SEP1*/*2*/*3*/*4*. These genes usually showed varying degrees of functional redundancy and subfunctionalization, which may be attributed to the different arrangement and spacing of the CArG-boxes in their *cis*-regulatory regions [42,43]. The expression pattern of the class E gene in *B*. *rapa* is different from that in *A*. *thaliana*. Previous studies on *A*. *thaliana* showed that *SEP1* and *SEP2* were expressed in four-whorled floral organs; *SEP3* was expressed in floral organs except for sepals, while *SEP4* was mainly expressed in sepals [44,45]. In this study, the *SEP1* lineage genes were extremely lowly expressed in *B*. *rapa* petals, while *SEP2* lineage genes were highly expressed in the pistil and ovule of *B*. *rapa* but lowly expressed in other floral organs. Moreover, *SEP3* lineage genes were expressed in floral organs except for stamens of *B*. *rapa*, while *SEP4* lineage genes were expressed in the pistil and sepals of *B*. *rapa* and highly expressed in the pistil. This difference in expression pattern can be observed in several plant species, such as petunia and tomato, which may result from flower morphological diversity [46]. The orthologs of E-*SEP1*, *SEP2,* and *SEP3* showed different expression patterns in the pistils of wt and *tetrac*. Part of the E-*SEP1* (*Bra008674*), *SEP2* (*Bra021470*) and *SEP3* (*Bra032814*) lineage genes was lowly expressed while the other E-*SEP1* (*Bra006322*), *SEP2* (*Bra039170*), and *SEP3* (*Bra010955*) lineage gene was highly expressed in the pistil of *tetrac*. This indicates that there is functional redundancy among these genes, as well as that subfunctionalization and neofunctionalization may occur between their homologous genes. The expression of *SEP4* in *tetrac* carpels decreased significantly, indicating that it is a negative regulatory gene for the development of carpels. In *tetrac* ovules, the expression level of *SEP1* and *SEP3* was significantly lower than that of wt, which indicated that these two genes negatively regulated the development of ovule number.

The results of cis-elements analysis revealed that hormone-responsive *cis*-elements are found in most ABCDE MADS-box genes. Multiple hormones are involved in ovule development and ovule number regulation via controlling pistil development, carpel fusion, and carpel margin meristem (CMM) formation, including auxin, brassinosteroid (BR), cytokinin (CK), and gibberellin (GA) [47]. Previous studies have shown that AG coordinates the termination of floral meristem (FM) and the establishment of gynoecium by regulating the crosstalk between CK and auxin. In the early developmental stages of A. thaliana, AG begins to repress the homeodomain transcription factor WUSCHEL (WUS) directly or indirectly through KNUCKLES (KNU), a regulator that encodes C2H2-type zinc proteins. Then, the repression of WUS is reinforced through different pathways, including the CRABS CLAW-TORNADO2 (CRC-TRN2) pathway. At the same time, AG and auxin activate ETTIN (ETT; an auxin response factor), inhibiting the expression of ISOPENTENYL TRANSFERASE (ipt), LONELY GUY (LOGs), and ARABIDOPSIS HISTIDINE KINASE 4 (AHK4), which reduces cytokinin activity [48]. To this end, the interaction between the ABCDE genes and hormones needs to be explored by additional experiments.

In addition, multilocular silique is considered as a trait associated with high yield in rapeseed, a few recent studies on the genetic regulation of the multilocular trait in *Brassica* crops have been reported. Yang et al. (2021) showed that the multilocular trait was monogenically governed by a recessive nuclear gene in *B. rapa* var. *srb*, and the candidate gene *Brclv3_Asp_*_12_ with a novel C-to-G single-nucleotide mutation in the core CLE motif of BrCLV3. Transgenic complementation studies and in vitro peptide assays further confirmed that the *Brclv3_Asp12_* allelic mutation in *srb* could lead to reduced activity of the CLV3 peptide, resulting in the formation of multilocular phenotype [49]. Xu et al. (2022) indicated the small G protein gene *BjROP10* was involved in regulating the formation of multilocular silique in *B. juncea*. In the T_0_ generation, 20 transgenetic-positive seedlings were obtained, 13 of which showed changes of the number of carpels [50]. These provide experimental basis and experimental materials for studying the molecular mechanism of the development of multilocular trait in rapeseed.

## 4. Materials and Methods

### 4.1. Plant Material and Experimental Treatments

Two pistil types of *B*. *rapa*, *tetrac*apel (*tetrac*) and wild-type (wt), were used in this study. The *tetrac* mutant was obtained from Professor Ba Dan, College of Plant Science, Tibet Agricultural and Animal Husbandry University. Seeds were sown in plastic seedling trays after germination, and the plants were grown under specific conditions (25/22 °C day/night temperature, 60–70% RH, with a 16 h photosynthetic photon flux density of 300 µmol·m^−2^·s^−1^) in a mixture composed of 6:3:1 peat soil:vermiculite:perlite. 

### 4.2. Measurement of Floral Organ Traits

To figure out the difference in the floral organs between *tetrac* and wt, a stereomicroscope (Nikon 90i, Tokyo, Japan) was used for observation. Measurements of the length and width of stigmas and stylus were taken by a vernier caliper. Thirty five flowers of each plant of *tetrac* and wt were randomly selected in this study, and the experiment was repeated three times. 

### 4.3. Selection of Target Genes and Chromosomal Location

The ABCDE genes in the MADS-box family were selected from the study of Saha, et al. [34], and the Genbank accession numbers of these genes are provided in Table S6. Although the latest genome of *B*. *rapa* accession (Brara_Chiifu_V3.5) has been released, it was abandoned because of its gene annotation errors (Figures S1–S4). Therefore, Brara_Chiffu_V1.5 was performed as the reference genome in this study. The chromosome lengths and gene locations were identified from the Brassica database (http://brassicadb.cn/) (accessed on 1 March 2023). TBtools [51] software (version 1.119) was used to draw a chromosome map of the selected genes.

### 4.4. Phylogenetic Analysis

The ABCDE proteins of *B*. *rapa* and *A*. *thaliana* were aligned using MAFFT software (version 7) [52], and the phylogenetic tree was constructed by the MEGA7 program using the Maximum Likelihood (ML) algorithm [53]. Bootstrap analysis with 1000 replicates was employed to evaluate the significance of the nodes. 

### 4.5. Gene Structure, Conserved Motif, and Cis-Elements Analyses

The gene structure of ABCDE genes in the MADS-box family was extracted from the general feature format (GFF) file generated from the Brassica database (Brara_Chiifu_V1.5). The online program MEME Suite 5.4.1 (https://meme-suite.org/meme/tools/meme) (accessed on 3 March 2023) was applied to analyze the conserved motifs in the ABCDE protein. Default parameters were selected, except that the maximum motif number was 8. The upstream sequences (2 kb) of CDS of ABCDE genes were extracted from the *B*. *rapa* genome, and the online program PlantCARE was used to predict and annotate cis-acting elements [54]. In addition, the program MEME Suite 5.4.1 was used to detect the conserved motifs of the promoter of target genes and compared them with the studied transcription factor binding site (TFBS) from the JASPAR CORE database via the motif comparison tool Tomtom (https://meme-suite.org/meme/tools/tomtom) (accessed on 3 March 2023), in order to identify transcription factors that may regulate the target genes. The gene structure, conserved motifs, and cis-elements were all visualized by TBtools software (version 1.119). 

### 4.6. RNA Extraction and Real-Time Quantitative PCR Assay (qPCR)

Total RNA was extracted using an RNA Extraction kit (Omega Bio-Tek, Shanghai, China), and the quality of total RNA was monitored with a Nanodrop spectrophotometer (Thermo Fisher Scientific, Inc, Waltham, MA, USA). cDNA was synthesized by 1 μg RNA using the PrimeScript™ RT reagent Kit with gDNA Eraser (Takara, Shiga, Japan) according to the manufacturer’s instructions. Primer 5.0 software was used to design primers and verify their specificity in the Brassica Database (http://brassicadb.cn/) (accessed on 4 March 2023). *BraUBC10* was used for the internal reference gene [55]. The primer sequence information is shown in Table S7. The qRT-PCR assays were conducted in a Bio-Rad CFX96 Real-Time PCR Detection System (Bio-Rad Laboratories, Inc., Hong Kong, China) using THUNDERBIRD^®^ SYBR^®^ qPCR Mix (Toyobo, Shanghai, China). The reaction mixture (15 µL in volume with 7.5 µL THUNDERBIRD SYBR qPCR mix, 0.3 µL forward primer, 0.3 µL reverse primer, 1 µL cDNA and 5.9 µL ddH_2_O) and the thermocycler conditions were based on the manufacturer’s protocol: predenaturation at 95 °C for 30 s; 40 cycles at 95 °C for 5 s and 60 °C for 30 s; and a melt cycle at 65 °C for 5 s. The qRT-PCR assays were performed using three biological and three technical replicates. The 2^−∆∆C^ value was used to measure the relative expression levels of specific genes [56], and the heatmap was drawn by TBtools (version 1.119) for tissue-specific analysis.

### 4.7. Statistical Analysis

Statistical analysis was performed using IBM SPSS Statistics 23 (SPSS Inc., Chicago, IL, USA). Student’s *t*-test was employed to determine significance, and differences were considered significant at *p* < 0.05 unless otherwise noted. All data shown represent the mean ± SD. 

## 5. Conclusions

This is a systemic analysis of the ABCDE genes in the MADS-box family, which showed their expression patterns in five floral organs and detected the expression levels of C, D, and E genes in the pistil and ovule of different pistil types in *B*. *rapa*. Our data confirm that class C, D and E genes are involved in the development of pistil and ovule. Class C genes positively regulate the development of carpel and ovule number, while class D and E genes might show various degrees of subfunctionalization and neofunctionalization. The differentially expressed genes described in this study might be exploited for the molecular mechanism of multilocular in *B. rapa*, and these results presented here are also helpful to select suitable candidate genes for improving the seed yield traits in currently cultivated rapeseed and vegetable varieties.

## Data Availability

All data in this study can be found in the manuscript or the Supplementary materials.

## Supplementary Materials

The following supporting information can be downloaded at: https://www.mdpi.com/article/10.3390/plants12112218/s1, Table S1: Measurement of female organs in two pistil types of *Brassica rapa*; Table S2: Motif sequences identified within the ABCDE class MADS-box genes in *B*. *rapa* using the MEME tool; Table S3: *Cis*-elements within 2-kb upstream of transcription start codon of ABCDE class MADS-box genes in *B*. *rapa*; Table S4: Functions of cis-elements in the promoters of ABCDE class MADS-box genes in *B*. *rapa*; Table S5: Potential transcription factor binding sites of ABCDE class MADS-box genes in *B*. *rapa*; Table S6: Genbank accession number of ABCDE genes in the MADS-box family in *B*. *rapa*; Table S7: qRT-PCR primers of ABCDE genes in the MADS-box family; Figure S1: Phylogenetic relationships of ABCED genes in *Arabidopsis thaliana* and *Brassica rapa* (Brara_Chiffu1.5 and V3.5); Figure S2: Phylogenetic relationships, conserved motif compositions and structural domains of *B. rapa* (Brara_Chiffu_V3.5) and *Arabidopsis* ABCDE class MADS proteins; Figure S3: The result of sequence alignment of 11 target genes between Brara_Chiffu_V1.5 and Brara_Chiffu_V3.5; Figure S4: The MADS domain in the upstream amino acid sequences of the CDS location of the target genes.
